# Tabular Handbook of Auscultation and Percussion

**Published:** 1879-07

**Authors:** 


*A Tabular Handbook of Auscultation and Percussion.* By HERBERT C. CLAPP, A. M., M. D. With four Plates. Boston: Houghton, Osgood and Company, 1879. Buffalo: Peter Paul and Bro.

Handbooks of Auscultation and Percussion, with Plates, have made the active physician familiar with Physical Diagnosis to a degree which could not have been otherwise obtained, as they have little time to devote to careful study of complete detail treatises upon the subjects so greatly simplified by these works, modestly termed by authors as "handbooks." The author of this work has divided his subject into eleven tables or chapters, which comprise a quite complete discussion of the facts which constitute the art of Physical Diagnosis as understood at the present time.

